# Association of candidate single nucleotide polymorphisms with somatic mutation of the epidermal growth factor receptor pathway

**DOI:** 10.1186/1755-8794-6-43

**Published:** 2013-10-23

**Authors:** Samuel Wormald, Liz Milla, Liam O’Connor

**Affiliations:** 1 Division of Systems Biology and Personalized Medicine, The Walter and Eliza Hall Institute of Medical Research, Melbourne, Australia; 2 Department of Medical Biology, The University of Melbourne, Melbourne, Australia; 3 VLSCI Life Sciences Computation Centre, The University of Melbourne, Melbourne, Australia

## Abstract

**Background:**

Tumour growth in colorectal cancer and other solid cancers is frequently supported by activating mutations in the epidermal growth factor receptor (EGFR) signaling pathway (Patholog Res Int 2011:932932, 2011). Treatment of metastatic colorectal cancer with targeted anti-EGFR therapeutics such as cetuximab extends survival in only 25% of patients who test wild-type for KRAS, while the majority of patients prove resistant (J Clin Oncol 28(7):1254–1261, 2010).

Prediction of cetuximab responsiveness for KRAS wild-type colorectal cancers is currently not well defined, and prognostic biomarkers would help tailor treatment to individual patients. Somatic mutation of the EGFR signalling pathway is a prevalent mechanism of resistance to cetuximab (Nature 486(7404):532–536, 2012). If the human genome harbours variants that influence susceptibility of the EGFR pathway to oncogenic mutation, such variants could also be prognostic for cetuximab responsiveness.

**Methods:**

We assessed whether patient genetic variants may associate with somatic mutation of the EGFR signalling pathway. We combined tumour mutation data from the Cancer Genome Atlas with matched patient genetic data, and tested for germline variants that associate with somatic mutation of the EGFR pathway (including EGFR, KRAS, BRAF, PTEN and PIK3CA).

**Results:**

Two single nucleotide polymorphisms (SNPs) located 90 kb upstream of the TERT oncogene associated with somatic mutation of the EGFR pathway beyond the threshold of genome-wide significance: rs7736074 (P = 4.64 × 10^-9^) and rs4975596 (P = 5.69 × 10^-9^). We show that allelic variants of rs7736074 and rs4975596 modulate TERT expression levels in multiple cancer types, and exhibit preliminary prognostic value for response to cetuximab.

**Conclusions:**

We have identified two germline SNPs that associate with somatic mutation of the EGFR pathway, and may be prognostic for cetuximab responsiveness. These variants could potentially contribute to a panel of prognostic biomarkers for assessing whether metastatic colorectal cancer patients are likely to derive benefit from cetuximab treatment. Genotyping of a large cohort of cetuximab-treated colorectal cancer patients is called for to further clarify the association.

## Background

The growth of solid tumours is frequently supported by aberrant expression of epidermal growth factor receptor (EGFR) or activating mutations in downstream signalling components [1]. Monoclonal antibodies directed against EGFR, including cetuximab and panitumumab, have shown efficacy both as monotherapies and in combination with chemotherapy for the treatment of colorectal cancer (CRC) [2]. Despite providing new avenues of treatment for solid cancers, effectiveness in the clinic has proved variable. 40% of CRC cases harbor an activating mutation in KRAS and derive no benefit from anti-EGFR therapy, while only 13% of KRAS wild-type cases show an objective response [3,4].

Regardless of their initial response, patients invariably develop resistance to targeted EGFR therapy [3,5,6]. Resistance is likely acquired by the emergence of mutations within EGFR or the EGFR pathway, including KRAS, BRAF, PIK3CA and PTEN. In KRAS wild-type CRC treated with cetuximab, 6 out of 10 cases acquire activating mutations in KRAS [3], and activating mutations in EGFR occur in 2 out of 10 cases [6]. Likewise, half of all non-small cell lung cancers treated with the EGFR inhibitors gefitinib or erlotinib acquire a second mutation in exon 20 of EGFR that confers resistance [5]. As response durations are typically measured in months, strategies to circumvent acquired drug resistance are needed.

The personalization of cancer care aims to predict effective therapy regimes according to the molecular profiles of individual patients and their cancers [7]. Germline SNPs in two components of the EGFR signalling pathway, EGF and Cyclin D1, are associated with overall survival in advanced CRC patients treated with cetuximab monotherapy [8], and a SNP in LIFR shows association with response to cetuximab combination therapy [9]. At the tumour level, somatic mutations in EGFR, KRAS, BRAF, PTEN and PIK3CA are associated with poor response to anti-EGFR therapy in CRC [2,10]. Even the majority of cancers initially negative for these mutations fail to respond [2], probably because subpopulations harboring drug-resistant mutations have been selected [3]. The identification of germline biomarkers that can predict whether a cancer is predisposed to activating mutations in the EGFR pathway would therefore be an extremely useful therapeutic tool.

## Methods

### Data sets

Germline SNP data (Affymetrix SNP 6.0) for cancer patients were obtained from The Cancer Genome Atlas (TCGA - level 2 Birdseed output) [11]. Matched somatic mutation data and RNA seq data were obtained from the TCGA exome sequencing pipeline and the TCGA RNA seq pipeline respectively. Where multiple replicate specimens were available from a single patient, one representative specimen was selected at random. For association analysis, patients were only included where both germline SNP data and matched somatic mutation data were available. For RNA-seq analysis, patients were only included where both germline SNP data and matched tumour RNA-seq data were available.

Germline SNP chip data for Korean colorectal cancer patients and matched *in vitro* cetuximab response levels [9] were obtained from the Gene Expresison Omnibus [12] (GSE21228).

### Genome wide association analysis

Genome wide SNP association was performed using the GWASTools package for R [13]. Associations were tested for using logistic regression under an additive model. For quality control, SNPs exhibiting > 5% missing genotype calls or non-Hardy-Weinberg equilibrium (p < 0.001) were excluded. A relatively high minor allele frequency cutoff of 10% was chosen due to the moderate number of patients and the high frequency of the measured outcome within the cancer patient population (meaning that rare SNPs are unlikely to prove informative). Non-autosomal SNPs were also excluded. In total, 580,710 out of 906,600 SNPs on the Affymetrix Human SNP 6.0 array were included in the final analysis. The genome-wide significance p-value cutoff was calculated as 0.05/(580,710 SNPs tested) = 8.61 × 10^-8^. Measurement of the genomic inflation factor (λ) and adjustment of P values for genomic inflation was performed using the genomic control functionality of the METAL [14] software package. Alternatively, eigenvectors as determined by EIGENSTRAT [15] were included as covariates in a linear regression model.

### RNA-Seq analysis of TERT expression levels

Raw counts from TCGA RNA-seq data were processed using edgeR [16]. Briefly, counts were normalized within samples, and negative binomial linear models applied, allowing gene-level variance to be quantified using Cox-Reid estimates of common and tagwise dispersions. Differential expression was then tested for using a generalized linear model likelihood ratio test.

## Results

### Genetic association with EGFR pathway status in cancer

We sought to determine whether a patient’s germline genetic profile influences susceptibility to mutation in EGFR or downstream signaling components. To approach this problem, we made use of The Cancer Genome Atlas (TCGA) [11] project which collects both somatic mutation data for patient tumours, as well as patients’ germline genetic profiles. Individual cancer types within TCGA comprise too few patients to attempt large scale association analysis, however as somatic mutation of the EGFR pathway is a hallmark of multiple types of solid cancer types, we sought to maximize the power of our study by combining patients across multiple cancer types that exhibit high frequency of mutation in the EGFR pathway. We note this increase in patient numbers comes at the expense of potentially losing signals specific to only single cancer types.

**Figure 1:**
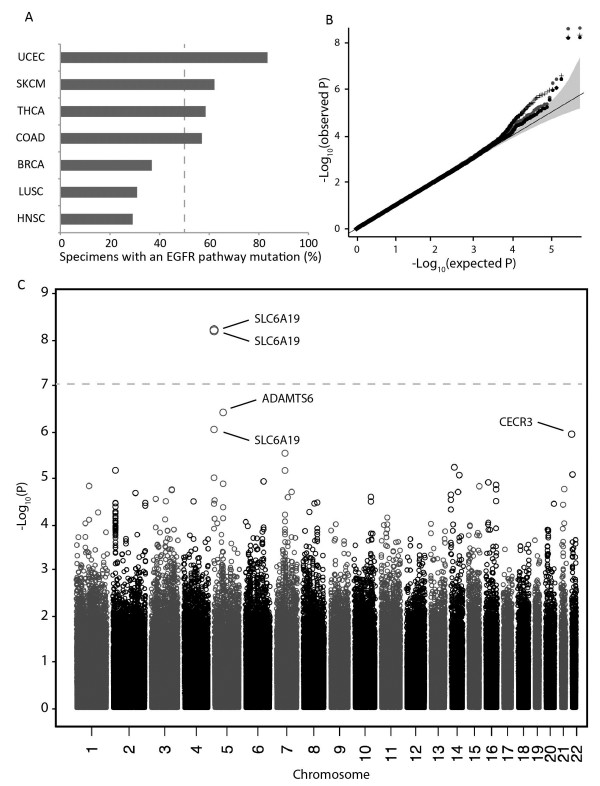
**Frequencies of somatic mutation in the EGFR pathway for different solid tumour types. (A)** TCGA tumour specimens were classified by mutation status for commonly mutated components of the EGFR pathway, including EGFR, KRAS, BRAF, PIK3CA and PTEN. The EGFR pathway was considered mutant if a non-synonymous mutation was detected in one or more of these factors. High and low mutation frequencies were determined by a 50% cutoff (dashed line). Cancer types with little or no evidence of EGFR pathway mutation are not shown. Somatic mutations for individual specimens were obtained from publicly available TCGA somatic mutation data (based on MutSig analysis of whole-exome sequencing). LUAD was excluded from the study due to reported strong environmental association between KRAS mutation status and smoke exposure [17]. **(B)** Quantile-quantile plot of GWAS SNP p-values for association with somatic mutation status of the EGFR pathway. Cancer types included in the analysis were: UCEC, SKCM, THCA and COAD. Black points show p-values adjusted for population stratification using genomic inflation control; crosses show p-values adjusted for population stratification by incorporation of the top 10 eigenvectors as covariates; grey points show non-adjusted p-values. **(C)** Manhattan plot of GWAS SNP p-values for association with somatic mutation status of the EGFR pathway. P-values were adjusted for genomic inflation. Grey dashed line indicates the genome-wide significance threshold (p = 8.78 × 10^-8^). Abbreviations: UCEC – uterine corpus endometrial carcinoma. SKCM – skin cutaneous melanoma. THCA – thyroid carcinoma. COAD – colorectal adenocarcinoma. BRCA - breast invasive carcinoma. LUSC - lung squamous cell carcinoma. HNSC – head and neck squamous cell carcinoma. LUAD – lung adenocarcinoma.

First, somatic mutation frequencies for commonly mutated components of the EGFR pathway (EGFR, KRAS, BRAF, PIK3CA and PTEN) were assessed in multiple solid cancer types (Figure 1A). Cancer types with moderate to high frequencies of non-synonymous mutations in the EGFR pathway (> 50%; Figure 1A) were combined for subsequent analysis: uterine corpus endometrial carcinoma (UCEC), skin cutaneous melanoma (SKCM), thyroid carcinoma (THCA) and colorectal adenocarcinoma (COAD). As each of these cancer types exhibits dependency on components of the EGFR pathway at similar frequencies (Figure 1A), we anticipated that through combining them we might uncover a common genetic predisposition.

Next, a genome-wide association study (GWAS) was performed to measure germline genetic association with somatic mutation of the EGFR pathway. Combining cases of UCEC, SKCM, THCA and COAD yielded a total of 1,013 patients (Table 1). From 569,429 SNPs across 1,013 germline samples, a low genomic inflation factor of 1.026 was measured, suggesting a minimal level of population stratification (Figure 1B).

**Table 1:** Classification of TCGA patients according to EGFR pathway mutation status

		**Number of patients**	
**Cancer type**	**EGFRpath +**	**EGFRpath -**	**Total**
THCA	187	135	**322**
UCEC	206	42	**248**
SKCM	131	88	**219**
COAD/READ	128	96	**224**
All	652	361	**1013**

*Abbreviations*: *THCA* thyroid carcinoma, *UCEC* uterine corpus endometrial carcinoma, *SKCM* skin cutaneous melanoma, *COAD* colorectal adenocarcinoma, *READ* rectal adenocarcinoma, EGFRpath + somatic mutation, *EGFRpath* - not mutated.

Two SNPs were detected beyond the genome-wide significance threshold P < 8.78 × 10^-8^: rs7736074 (P = 4.64 × 10^-9^) and rs4975596 (P = 5.69 × 10^-9^; see Figure 1C, Table 2). These SNPs, located on the short arm of chromosome 5 and separated by 109 bp, show a high degree of linkage disequilibrium, with R^2^ = 1.0 for individuals of northern-European ancestry, and R^2^ = 0.935 for individuals of Chinese or Japanese ancestry [18]. The close linkage between rs7736074 and rs4975596 provide important validation that the observed association is not a technical artifact of the SNP genotyping platform.

**Table 2:** Top 5 SNPs identified by GWAS for EGFR pathway status

**Gene**	**SNP ID**	**Chromosome**	**Position**^**a**^	**Region**	**P value**^**b**^
SLC6A19	rs7736074	5	1189456	Upstream	4.64 ×10^-9^
SLC6A19	rs4975596	5	1189347	Upstream	5.69 ×10^-9^
ADAMTS6	rs715676	5	64450473	Intron	1.56 ×10^-6^
SLC6A19	rs6554634	5	1186121	Upstream	1.76 ×10^-6^
CECR3	rs4819993	22	17761425	Upstream	3.77 ×10^-6^

^a^hg19 coordinates.

^b^Adjusted for genomic inflation.

An additional quality control measure, principle component analysis, indicated that mutation status of the EGFR pathway is not simply driven by population structure (Additional file 1: Figure S1). Furthermore, different approaches of accounting for population structure did not dramatically alter the p-values for rs7736074 and rs4975596 (Figure 1B and Additional file 1: Figure S2). At the probe level, genotype intensity groups are generally well defined (Additional file 1: Figure S3), however we identified some samples with genotype call p-values above 0.05 (4% for rs7736074, 7% for rs4975596), indicating lower confidence calls (Additional file 1: Figure S4). Both SNPs remain beyond genome-wide significance with these lower-confidence calls excluded from the analysis (rs7736074: 9.46×10^-9^; rs4975596: 2.67 × 10^-8^).

Rs7736074 and rs4975596 are located approximately 12 kb upstream of SLC6A19, and 90 kb downstream of the gene encoding telomerase reverse transcriptase (TERT). Genetic variants near TERT are strongly associated with predisposition to eight or more different cancer types [19], suggesting a potential mechanism by which rs7736074 and rs4975596 could influence the oncogenic potential of the EGFR signaling pathway through modulation of TERT activity.

**Figure 2:**
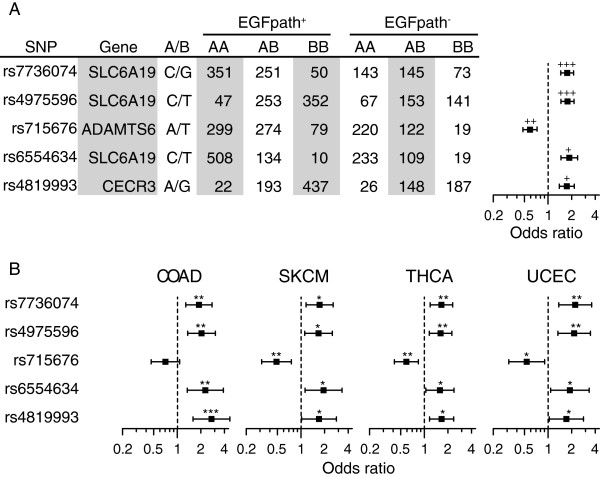
**Patient genotype numbers and odds ratios for the top five SNPs associated with EGFR pathway status combined across four cancer types (COAD, SKCM, THCA and UCEC). (A)** Odds ratios for combined analysis. **(B)** Odds ratios for individual cancer types. Error bars indicate 95% confidence intervals. ^+++^P < 5 × 10^-9^; ^++^P < 5 × 10^-7^; ^+^P < 5 × 10^-6^; ^***^P < 5 × 10^-4^; ^**^P < 5 × 10^-3^; ^*^P < 0.05.

We also identified three additional SNPs that appear suggestive based on visual inspection of the quantile distribution for SNP P values, despite failing to achieve genome-wide significance (Figure 1B). Of the top five SNPs identified, four were located on chromosome 5, and one on chromosome 22 (Table 2). Uniform effect sizes were observed both for the combined analysis (Figure 2A) and for individual cancer types (Figure 2B).

### SNPs rs7736074 and rs4975596 associate with TERT expression levels

**Figure 3:**
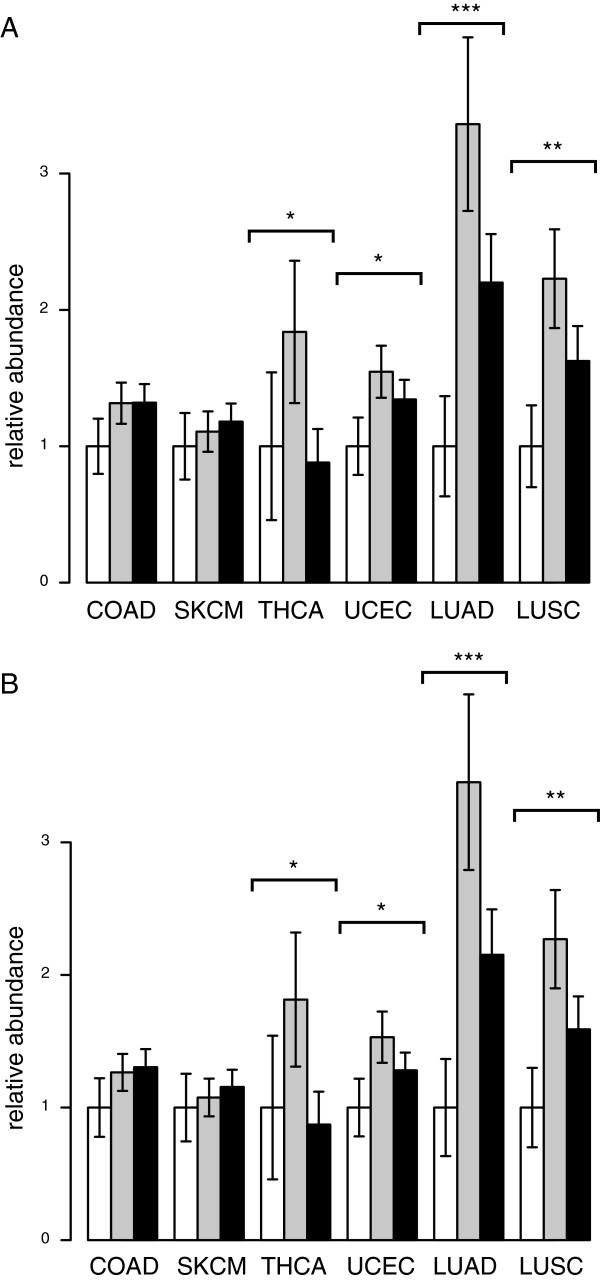
**Relative TERT mRNA abundance between genotypes in COAD, SKCM, THCA, UCEC, and two types of NSCLC: LUAD and LUSC. (A)** rs7736074: G/G (white bars), G/C (grey bars), C/C (black bars). **(B)** rs4975596: C/C (white bars), C/T (grey bars), T/T (black bars). RNAseq data was obtained for TCGA tumour specimens corresponding to patients whose germline SNP profiles were analysed in Figure 1. Significant differences in mRNA abundance between genotypes were calculated using edgeR [16] (see Methods). Error bars show standard error of the mean, and were derived from the edgeR dispersion metric. ^***^P < 5 × 10^-4^; ^**^P < 5 × 10^-3^; ^*^P < 0.05.

The 15p5.33 locus, harboring SLC6A19 and TERT, is of particular importance in non-small cell lung cancer (NSCLC), where copy number amplification of the region is found in 78% of cases [20]. As SNPs at this locus could potentially influence oncogenesis by modulating TERT expression, we examined whether rs7736074 and rs4975596 associate with TERT expression levels in COAD, SKCM, THCA, UCEC, as well as for two subtypes of NSCLC (squamous cell or adenocarcinoma). We detected modest significant differences (P < 0.05) in TERT expression between genotypes for THCA and UCEC, and larger significant differences for the two NSCLC subtypes (P < 0.005 and P < 0.0005; Figure 3A and 3B). We observed similar trends for COAD and SKCM, however these effects were not significant. We hypothesize that the observed differences between cancer types may be attributable to differing degrees of cellular heterogeneity, or to differing degrees of TERT sensitivity.

TERT expression profiles for rs7736074 and rs4975596 were nearly identical, reflecting the high degree of linkage between these polymorphisms (Figure 3A and 3B). The relationship between genotype and TERT expression was generally consistent between cancer tumour types (Figure 3A and 3B). Heterozygotes typically exhibited heightened expression levels, suggesting a complex relationship between genotype and other factors (such as copy-number or methylation) in determining TERT expression levels. In particular, the substantial differential expression of TERT between genotypes in the two NSCLC subtypes suggests that genotype could play a role in determining copy-number amplification of TERT.

### SNPs rs7736074 and rs4975596 associate with *in vitro* tumor sensitivity to cetuximab

**Figure 4:**
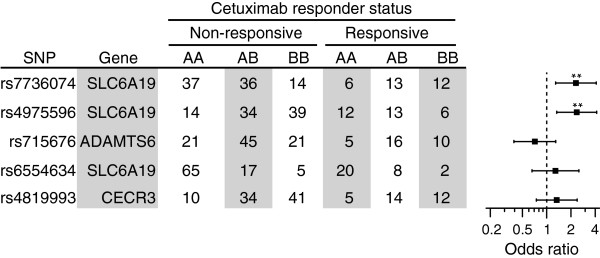
**SNPs rs4975596 and rs7736074 associate with *in vitro *tumour sensitivity to cetuximab.** Patient genotypes and odds ratios were calculated for *in vitro* response to cetuximab, using the same panel of SNPs as in Figure 2. Genotype and response data were obtained for 118 Korean colorectal cancer patients from the GEO database (accession GEO 21228) as described previously [9].

Understanding how germline genetic variation influences the EGFR pathway in cancer may aid in prediction of patient responses to targeted therapeutics. To test this hypothesis, and verify our GWAS findings in an independent population, we examined the association of SNPs from Table 2 with *in vitro* tumor response to cetuximab (Erbitux) using publicly available SNP data for 118 Korean colorectal cancer patients [9] (GEO series GSE21228; association with Erbitux response in the absence of chemotherapy). The profile of odds ratios for *in vitro* response to cetuximab (as measured by tumor cell viability) was concordant with the odds ratios observed for somatic mutation of the EGFR pathway (Figure 4; compare with Figure 2B). Significant association with *in vitro* cetuximab response was observed for SLC6A19 SNPs rs7736074 and rs4975596 (P = 0.003 and 0.002 respectively). This finding supports our hypothesis that SNPs predictive of EGFR pathway mutation status may serve as informative biomarkers for predicting cetuximab response.

## Discussion

At the molecular level, most human cancers can be classified into one or more subtypes of disease. The germline genetic profile of a patient can influence predisposition to specific cancer subtypes; in breast cancer, for example, FGFR2 variants are strongly associated with ER-positive but not ER-negative breast cancer [21]. In CRC, outgrowth of tumour subpopulations harboring mutations in components of the EGFR pathway is strongly associated with acquired resistance to cetuximab [4]. Cancer heterogeneity may confound the detection of such mutations by biopsy, or they may arise during the course of treatment. This study aimed to determine whether specific germline genetic factors may predispose patients to the acquisition of mutations in the EGFR pathway, and thus to cetuximab resistance. By including multiple components of the EGFR pathway in our association analysis, we aimed to isolate genetic variants that influence the EGFR pathway as a whole, as we reasoned these would likely be most informative.

We identified germline SNPs at 15p5.33 that associate with somatic mutation of the EGFR signaling pathway in TCGA patients. In an attempt to further validate this finding, we examined association of the SNPs with *in vitro* resistance to cetuximab (which likely reflects to some extent the mutation status of the EGFR pathway) in an independent cohort of CRC patients, and found them to be significant.

15p5.33 is a hotspot of genetic predisposition for multiple cancer types, probably because oncogenesis and cell immortalization are closely linked with the telomere maintenance activities of TERT [19]. We postulate that the SNPs we identified may be in linkage with a regulatory element that modulates TERT expression. Consistent with this hypothesis, we found TERT mRNA expression levels to be associated with genotype at rs4975596/rs7736074 in multiple cancer types. Association was strongest in squamous-cell carcinomas and adenocarcinomas of the lung, where the 15p5.33/TERT locus is amplified at particularly high frequency [20]. The other cancer types we examined exhibited similar regulatory trends albeit at decreased magnitude and significance, possibly due to differences in TERT dependence, tumour heterogeneity, or the action of alternative regulatory pathways at rs4975596/rs7736074 in lung cancer.

Numerous studies have reported regulation of TERT by EGFR-responsive factors including Wnt/B-catenin [22], Myc [23], and NFkB [24]. Further evidence for a regulatory link between EGFR and TERT was reported recently in malignant glioma, where 92% of cases harboring EGFR amplification were accompanied by a mutation in the TERT promoter [25]. Polymorphisms that disrupt a regulatory element linking EGFR signaling to TERT expression would thus impede the oncogenic potential of the EGFR pathway, and may reduce the likelihood of the pathway succumbing to somatic mutation.

## Conclusion

The EGFR pathway induces pro-proliferative and anti-apoptotic signals, and constitutes a convenient target for somatic mutation in cancer. The occurrence of such a mutation can impede the effectiveness of anti-EGFR therapeutics such as cetuximab. We used TCGA patient data to assess whether genetic variants may predispose to somatic mutation of the EGFR pathway. We identified two SNPs located 90 kb upstream of TERT, rs7736074 and rs4975596, that associate with EGFR pathway mutation (P < = 5.69 × 10^-9^). We found the same two SNPs were also predictive of *in vitro* cetuximab resistance using publicly available genetic data from Korean colorectal cancer patients [9]. Our results suggest that genetic variants may predispose to somatic mutation of the EGFR pathway, and consequently to resistance with anti-EGFR therapeutics. Larger studies are called for to further characterize the contribution of patient genetic variation to anti-EGFR therapeutic resistance.

## Competing interests

The authors declare they have no competing interests.

## Authors’ contributions

SW designed the study, designed and performed the analysis, analysed results, and prepared the manuscript. LM processed data, assisted with the analysis, and helped write the manuscript. LO assisted with the analysis, discussed results, and helped write the manuscript. All authors read and approved the final manuscript.

## Pre-publication history

The pre-publication history for this paper can be accessed here:

http://www.biomedcentral.com/1755-8794/6/43/prepub

## Supplementary Material

Additional file 1 This file comprises Supplementary Figures S1-S4. Click here for file
