# Supplementary material for: Association of candidate single nucleotide polymorphisms with somatic mutation of the epidermal growth factor receptor pathway
Source: BMC Med Genomics. 2013 Oct 23;6:43. doi: 10.1186/1755-8794-6-43 (PMC4016273; doi:10.1186/1755-8794-6-43)
Supplement: Additional file 1 — This file comprises Supplementary Figures S1-S4. [file 1755-8794-6-43-S1.pdf]

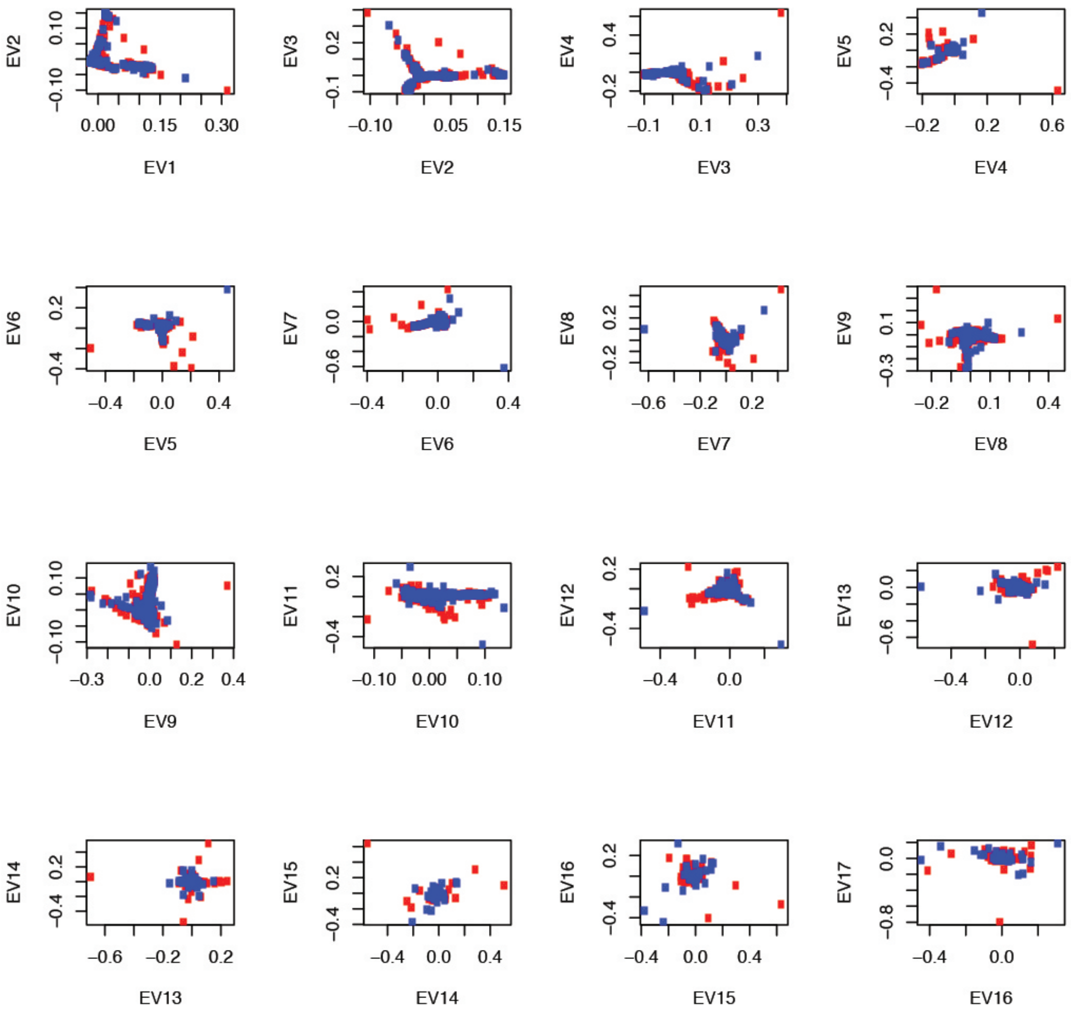

Supplementary Figure S1.
Pairwise plots of the first 16 eigenvectors from principal component analysis of genotypes for 1019 TCGA patients. Red points: patients harboring a somatic mutation in the EGFR pathway. Blue points: patients lacking a detectable mutation in the EGFR pathway.

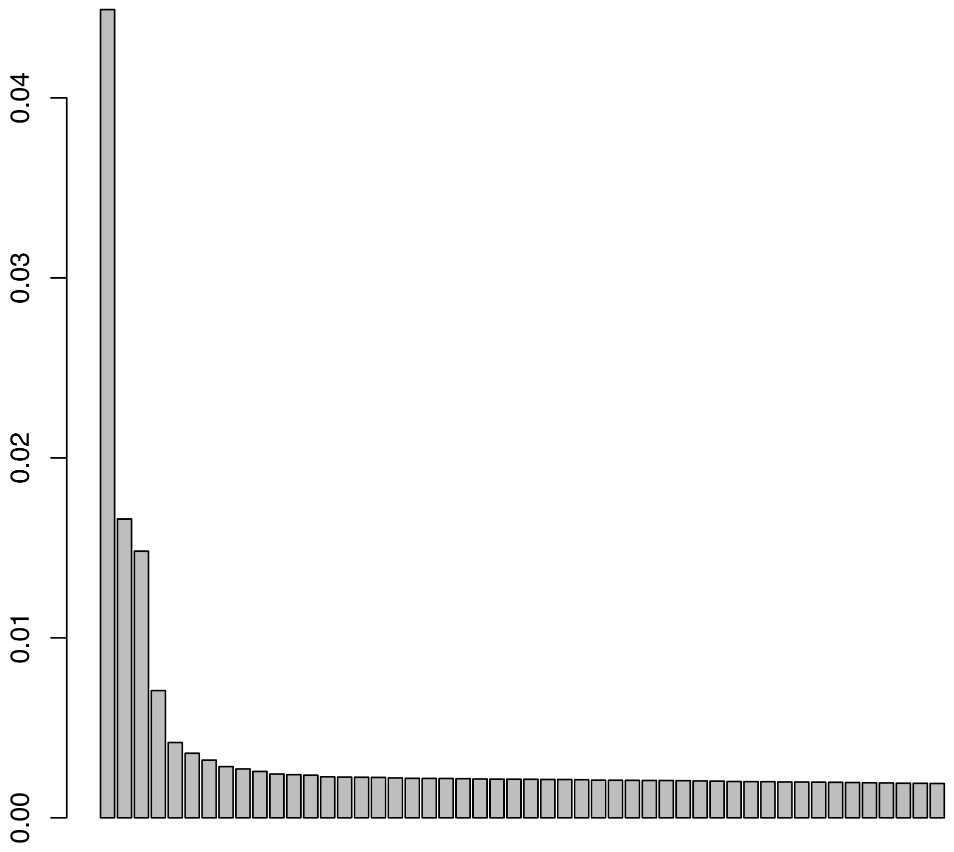

Supplementary Figure S2.
Scree plot of first 50 eigenvalues from principal component analysis of genotypes for 1019 TCGA patients (values shown as fraction of total). The first 10 were used as covariates for linear regression.

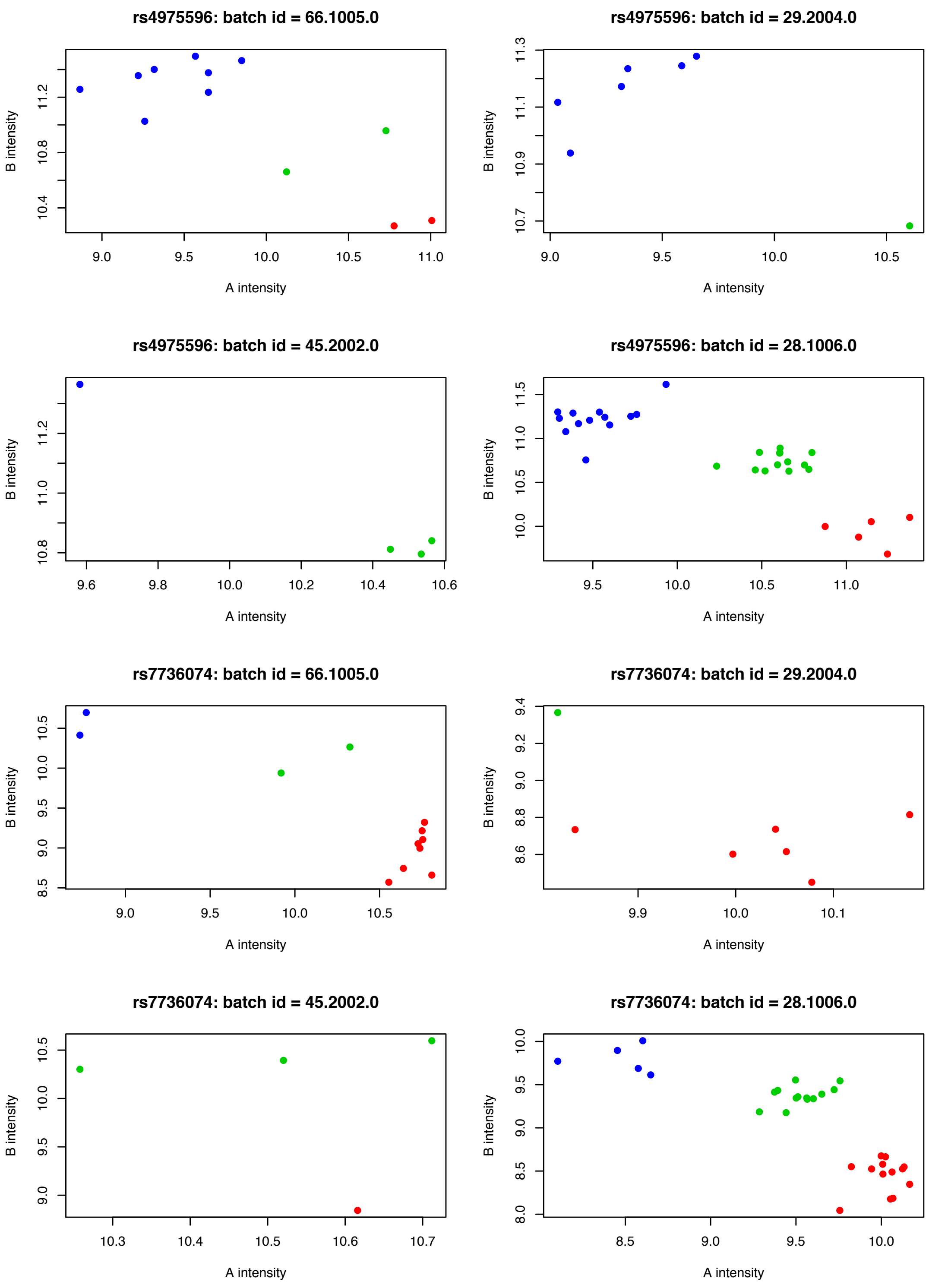

Supplementary Figure S3
SNP probe intensity plots of (A) rs7736074 and (B) rs4975596 for four arbitrary patient batches. Red: AA; Green: AB; Blue: BB.

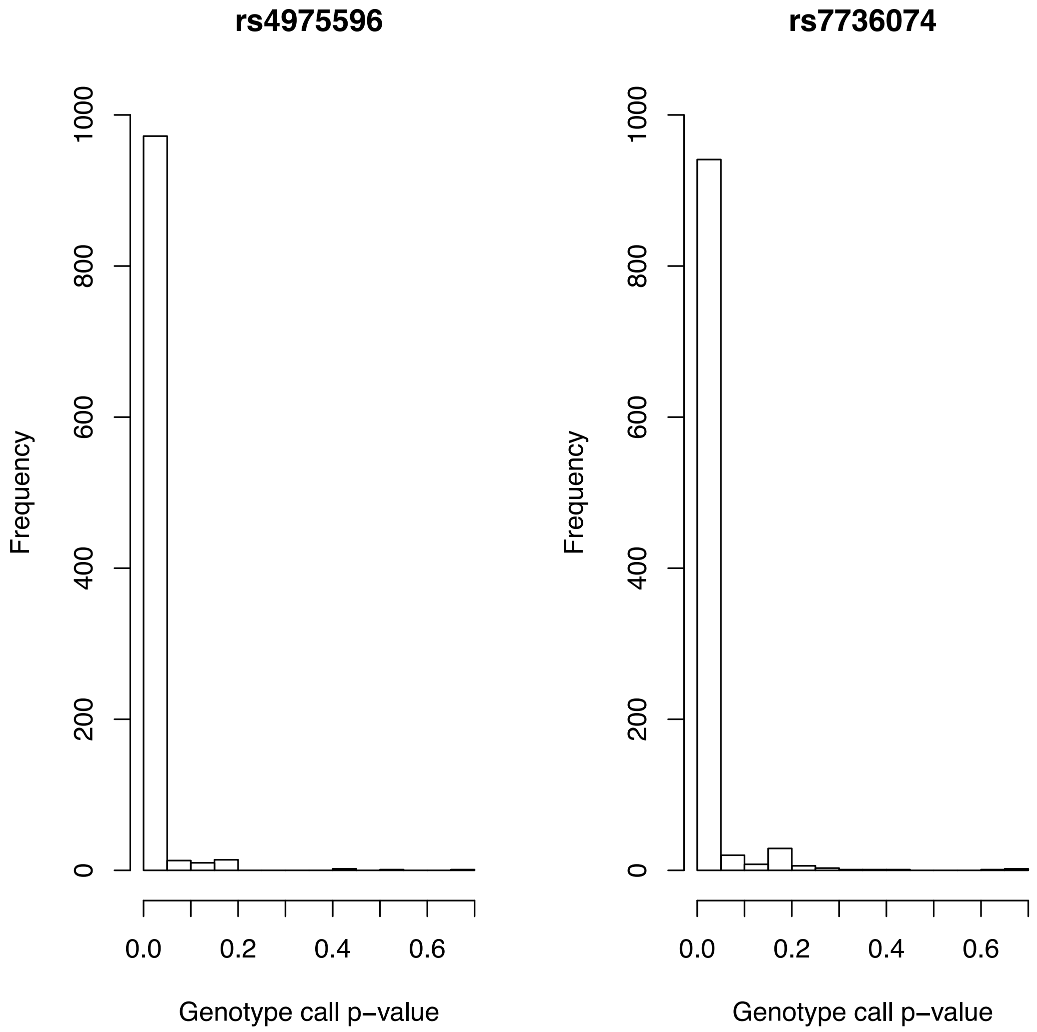

Supplementary Figure S4
Histograms of Birdseed genotype call p-values for rs4975596 and rs7736074.
